# Alpha-glucosidase inhibitors and risk of cancer in patients with diabetes mellitus: a systematic review and meta-analysis

**DOI:** 10.18632/oncotarget.17515

**Published:** 2017-04-28

**Authors:** Yiming Zhao, Yongjian Wang, Hanyu Lou, Lizhen Shan

**Affiliations:** ^1^ Department of Endocrinology and Metabolism, the Second Affiliated Hospital Zhejiang University College of Medicine, Hangzhou, Zhejiang, China

**Keywords:** alpha-glucosidase inhibitor, diabetes mellitus, cancer risk, meta-analysis, systematic review

## Abstract

Several studies have shown that anti-diabetic medications may modify the risk of cancer. We performed a systematic review and meta-analysis to evaluate the effect of alpha-glucosidase inhibitors (AGIs) on the risk of cancer in patients with diabetes mellitus. We conducted a systematic search of Medline, EMBASE, and Web of Science databases, up to September 30, 2016. Random-effects model was used to estimate the summary odds ratios (ORs) with 95% CI. Twenty-five studies (14 cohort, 7 case-control, and 4 randomized controlled trials) involving 1,285,433 patients with diabetes were included. Meta-analysis of observational studies showed that the use of AGIs was associated with a lower risk of developing cancer (OR = 0.86, 95% CI 0.78-0.96), especially gastrointestinal cancer (OR = 0.83, 95% CI 0.71-0.97). There was considerable heterogeneity across the studies introduced partly by the quality of included studies and adjustment for potential confounders. Meta-analysis of randomized controlled trials did not reveal any significant association between AGIs and cancer risk. Meta-analysis of observational studies indicated that AGIs may decrease the risk of cancer in individuals with diabetes.

## INTRODUCTION

Diabetes mellitus (DM) is a prevalent disease associated with considerable global health burden [1]. The number of patients with DM has significantly increased in the past few decades globally, especially in China [2, 3]. As DM is considered a risk factor for several types of cancer [4, 5], anti-diabetic medications (ADMs) have the potential to modify the risk of cancer [6, 7]. Metformin, an ADM, has been shown to exert antineoplastic effects through both insulin-dependent and insulin-independent mechanisms [8, 9]. However, data from various studies analyzing the anticancer effects of other types of ADMs, such as insulin, sulfonylureas, thiazolidinediones, alpha-glucosidase inhibitors (AGIs), dipeptidyl peptidase-4 inhibitors, glinides, glucagon-like peptide-1 (GLP-1) agonists, and sodium glucose cotransporter type 2 inhibitors, are not consistent [7, 10–14]. AGIs such as acarbose, voglibose, and miglitol are commonly used oral hypoglycemic agents in China and other eastern Asian countries. The association between AGI and risk of cancer has been inconsistently reported.

A meta-analysis of 13 studies (6 case-control studies, 2 cohort studies, and 5 randomized controlled trials [RCTs]) published in 2015 concluded that AGI was associated with a significantly higher risk (10%) of cancer [7]. The meta-analysis noted a significant risk in the case-control studies, but not in the cohort studies or RCTs, compared with risk in the control population [7]. However, the meta-analysis had several limitations such as a mixture of studies with observational studies and RCTs; a mixture of studies with different tumor types; small case numbers in most studies; and lack of differentiation between cases of type 1 and type 2 diabetes. Studies published after this meta-analysis also yielded inconsistent results. Most recently, a cohort study of DM patients in Taiwan showed that acarbose use reduced the risk of incident colorectal cancer in patients with diabetes in a dose-dependent manner [15], but a cohort study performed in Italy did not find any association between AGI use and cancer risk [16].

Our objective was to conduct a systematic review and meta-analysis of observational studies and RCTs to investigate the effect of AGI use on cancer risk in patients with DM.

## RESULTS

A total of 1399 unique studies were identified using the search strategy, of which 25 studies involving 1,285,433 patients with DM fulfilled the inclusion criteria and were pooled in the meta-analysis (14 cohort, 7 case-control, and 4 RCTs) [15–39].

### Study characteristics

The study characteristics are shown in Table 1. The earliest study period began in 1989 and the latest period ended in 2015. Fifteen of these studies were population-based studies, and the remaining 10 were hospital-based studies. Nineteen studies were from Asia and 5 studies were from Europe. One RCT was multicenter trial and included mixed populations (from China, Romania, and Spain). A majority of the included studies (22 studies) demonstrated cancer risk in patients with type 2 DM, and the remainders did not report the subtype of DM. Two studies assessed the modification of cancer risk with duration and dose of exposure to AGI [15, 34].

**Table 1:** Characteristics of included studies assessing the risk of cancer in patients with DM on AGI

Study	Design	Location / setting	Time period; mean F/U (years)	Sample size (AGI/total)	Type of cancer	Type of DM	Mean duration of DM (years)	Mean age	Type of AGI	Exposure ascertainment	Outcome ascertainment	Potential confounders
Nakamura, T[17]	RCT	Japan; HB	NR; 1	15/45	NR	2	16.8	55.5	voglibose	RCT	Adverse event review	NR
Pan, C[18]	RCT	China, Romania, Spain; HB	NR; 0.5	220/661	NR	2	1.3	51.9	Acarbose	RCT	Adverse event review	NR
Kawaguchi, T[19]	C-C	Japan; HB	2004.1-2008.12; NR	40/241	Liver	2	NR	68.8	NR	HDMS	Medical record review	NR
Yang, X[20]	Cohort	Hong Kong; HB	1996.12.1-2005.1.9; 4.9	829/6103	NR	2	6	57	Acarbose	Medical record review	ICD-9	NR
Bosco, J. L[21]	C-C	Denmark; PB	1989-2008; NR	204/4323	Breast	2	NR	NR	NR	Prescription databases	ICD-8 or ICD-10	NR
Monami, M[22]	C-C	Italy; HB	1998-2007, 6.3	8/482	NR	2	8.4	68.9	Acarbose	Medical record review	ICD-9	NR
Tseng, C. H.[23]	Cohort	Taiwan; PB	2003.1-2005.12; 3	NR/115731	Bladder	2	NR	NR	Acarbose	NHI database	ICD-9	1, 2, 3, 7
Tseng, C. H.[24]	Cohort	Taiwan; PB	2003.1-2005.12; 3	NR/52131	Prostate	2	NR	NR	Acarbose	NHI database	ICD-9	1, 2, 3, 7
Chang, C. H.[25]	C-C	Taiwan; PB	2000.12.31-2007.12.31; 7.9	3207/40969	Colon, Liver	2	New-onset	NR	NR	Pharmacy prescription database	ICD-9	NR
Kawamori, R.[26]	RCT	Japan; HB	2008-2010; 0.5	162/561	NR	2	NR	58.5	voglibose	RCT	Adverse event review	NR
Lai, S. W.[28]	Cohort	Taiwan; PB	2000-2008; 9	4638/19624	Lung	2	New-onset	56.4	NR	NHI database	ICD-9	1, 2, 3
Lai, S. W.[27]	Cohort	Taiwan; PB	2000-2008; 9	4449/19349	Liver	2	New-onset	56.4	NR	NHI database	ICD-9	1, 2, 3
Liao, K. F.[29]	Cohort	Taiwan; PB	1998–2007; NR	12301/49803	Pancreas	2	New-onset	55.9	NR	NHI database	ICD-9	NR
Tseng, C. H.[30]	Cohort	Taiwan; PB	1996-2005; NR	NR/115928	Thyroid	2	NR	NR	NR	NHI database	ICD-9	1, 2, 3, 6, 7
Chen, Y. L.[31]	Cohort	Taiwan; PB	2000-2008; 5.5	4472/19625	Gastric	NR	New-onset	56	Acarbose	NHI database	ICD-9	1, 2
Chiu, C. C.[32]	Cohort	Taiwan; PB	2000-2007; 7	2918/39515	Colon, Esophagus, Gastric, Rectum, Pancreas, Liver	NR	New-onset	58.52	NR	NHI database	ICD-9	1, 2, 3
Origasa H,[33]	C-C	Japan; HB	2005-2011; NR	26/95	Bladder	2	NR	69	NR	Medical record review	Medical record review	1, 6
Simo, R.[34]	C-C	Spain; HB	2008-2010; NR	115/2438	NR	2	6.4	72.0	NR	Pharmacy prescription database	ICD-10	1, 4, 5, 6, 8
Chen, Y. C.[35]	Cohort	Taiwan; PB	1998-2007; 2.5	150/7325	NR	2	New-onset	62.6	Acarbose	NHI database	ICD-9	1, 2, 3, 7
Lin, C. M[36]	Cohort	Taiwan; PB	2005–2010; NR	NR /34823	Lung, Liver, Colorectal, Breast, Oral cavity, Pancreas	2	New-onset	54.26	NR	NHI database	ICD-9	1, 2, 3, 6
Son, J. W.[37]	RCT	Korea; HB	2008.2-2009.1; 0.3	81/156	Gastric	2	12.2	56.1	voglibose	RCT	Adverse event review	NR
Tseng, Y. H.[15]	Cohort	Taiwan; PB	1998-2010; 3.4	199296/398592	Colorectal	NR	New-onset	54.1	Acarbose	NHI database	ICD-9	1, 2, 3, 6, 7
Valent, F.[16]	Cohort	Italy; PB	2002-2014; NR	NR/109255	Esophagus, Gastric, Colon, Rectum, Liver, Pancreas	2	NR	NR	NR	Pharmaceutical prescription database,	ICD-9	1, 2, 6
Dabrowski, M.[38]	C-C	Poland; HB	1998–2015; NR	32/406	NR	2	10.7	67.1	Acarbose	Medical record review	Medical record review	3, 4, 6, 7, 8
Tseng, C. H[39]	Cohort	Taiwan; PB	1998-2009, NR	47734/247252	Kidney	2	New-onset	NR	Acarbose	NHI database	ICD-9	NR

RCT, randomized controlled trial; C-C, case-control; PB, population based; HB, hospital based; DM, diabetes mellitus; F/U, follow-up; ICD-8/9/10, International Classification of Diseases, Eighth/Ninth/Tenth Revision; NR, not reported; NHI, National Health Insurance in Taiwan; HDMS, HCV-related diabetes mellitus study in Japan.

Potential confounders: 1 - age; 2 - sex; 3 - comorbidities; 4 - smoking; 5 - alcohol; 6 - diabetes status, including DM duration, DM control, other ADM use; 7 - social status, including living region, occupation, and income; 8 - BMI.

### Quality of included studies

The median Newcastle-Ottawa quality score for the observational studies was 7 (range 5-8). Table 2 depicts the methodological quality of all observational studies. The quality of the RCTs was moderate (Figure S1). The overall methodological quality of this body of evidence was moderate to high.

**Table 2:** Assessment of quality of included studies using Newcastle-Ottawa Scale

Cohort study										
	Selection				Comparability	Outcome				
Study	Representativeness of the exposed cohort	Selection of the non-exposed cohort	Ascertainment of exposure	Demonstration that the outcome of interest was not present at start of the study	Comparability of cohorts on the basis of design or analysis	Assessment of outcome	Was follow-up long enough for the outcome to occur?	Adequacy of follow up of cohorts	Total stars	Risk of bias
Yang, X[20]	*	*	*	*	*	*	*	-	7	Low
Tseng, C. H.[23]	*	*	*	*	**	*	-	*	8	Low
Tseng, C. H.[24]	*	*	*	*	**	*	-	*	8	Low
Lai, S. W.[28]	*	*	*	*	**	*	*	-	8	Low
Lai, S. W.[27]	*	*	*	*	**	*	*	-	8	Low
Liao, K. F.[29]	*	*	*	*	*	*	*	-	7	Low
Tseng, C. H.[30]	*	*	*	*	**	*	-	-	7	Low
Chen, Y. L.[31]	*	*	*	*	**	*		-	8	Low
Chiu, C. C.[32]	*	*	*	*	**	*	*	-	8	Low
Chen, Y. C.[35]	*	*	*	*	**	*	-	*	8	Low
Lin, C. M[36]	*	*	*	*	**	*	-	-	7	Low
Tseng, Y. H.[15]	*	*	*	*	**	*	-	-	7	Low
Valent, F.[16]	*	*	*	*	*	*	-	-	6	Moderate
Tseng, C. H[39]	*	*	*	*	-	*	-	-	5	Moderate
**Case-control study**										
	**Selection**				**Comparability**	**Exposure**				
**Study**	**Is the case definition adequate?**	**Representativeness of the cases**	**Selection of controls**	**Definition of controls**	**Comparability of cases and controls on the basis of design or analysis**	**Ascertainment of exposure**	**Same method of ascertainment for cases and controls**	**Non-response rate**	**Total stars**	**Risk of bias**
Kawaguchi, T[19]	*	*	-	*	-	*	*	-	5	Moderate
Bosco, J. L[21]	*	*	*	*	*	*	*	-	7	Low
Monami, M[22]	*	*	-	*	*	*	*	-	6	Moderate
Chang, C. H.[25]	*	*	-	*	*	*	*	-	6	Moderate
Origasa H,[33]	*	-	-	*	*	*	*	-	5	Moderate
Simo, R.[34]	*	-	*	*	*	*	*	-	6	Moderate
Dabrowski, M.[38]	*	-	-	*	**	*	*	-	6	Moderate

### AGI and the risk of any cancer

Of the 25 studies (21 observational and 4 RCTs) that reported on the association between AGI use and cancer risk, 4 demonstrated a decreased risk of cancer with AGI use [15, 28, 31, 36], 1 showed an increased risk [25], and 20 reported no significant relationship [16–24, 26, 27, 29, 30, 32–35, 37–39]. A meta-analysis of the observational studies demonstrated that AGI use (as compared with nonuse) was associated with a statistically significant 14% reduction in cancer incidence (*n* = 21 studies; odds ratio [OR] = 0.86, 95% CI 0.78-0.96) (Figure 2). There was considerable heterogeneity between studies (Cochran Q test *P* < 0.01; *I*^2^ = 82.4%). Of the study characteristics assessed in meta-regression, the quality of study and adjustment for potential confounders were statistically significant (*P* < 0.01) (Table 3). Meta-regression analysis did not show any significant effect size modification by other specific study characteristics considered, such as study design, setting, location, or duration of DM.

**Figure 1:**
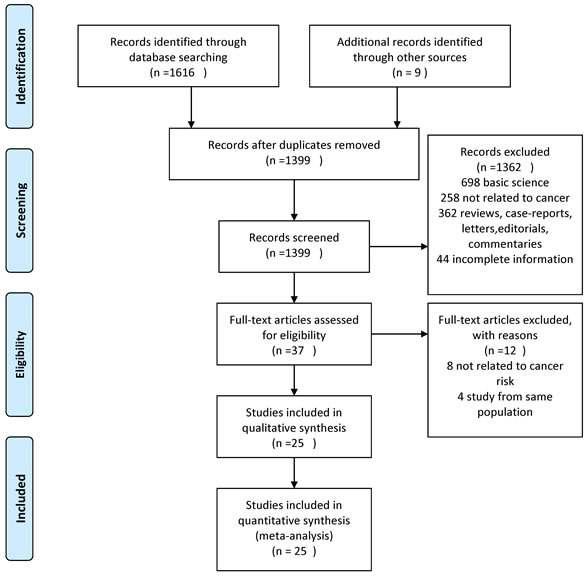
Study flow diagram in line with Preferred Reporting Items for Systematic Reviews and Meta-Analyses (PRISMA) recommendations

**Figure 2:**
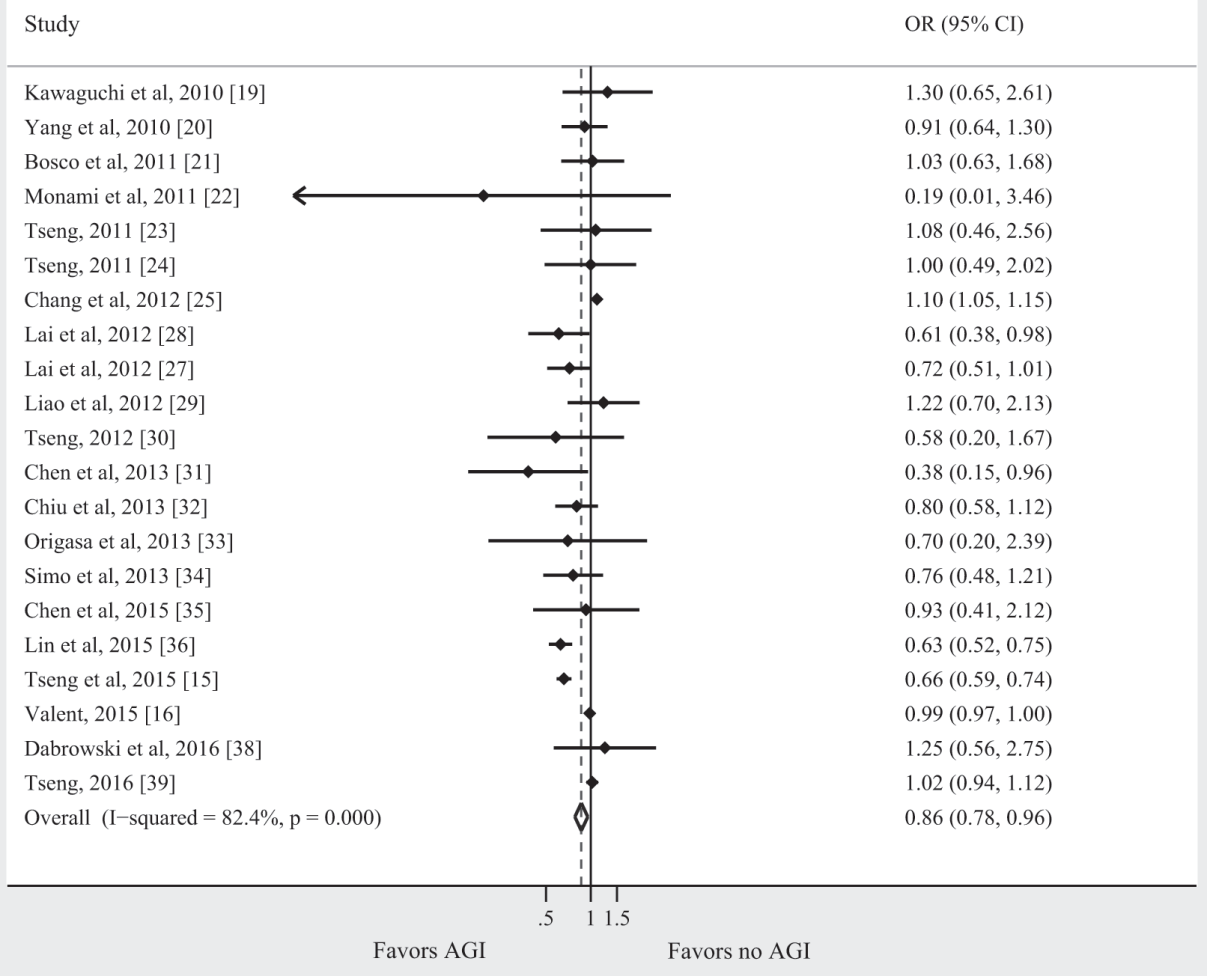
Summary of OR of observational studies assessing the risk of cancer with AGI use

**Table 3:** Subgroup analysis

Subgroups	No. of studies	OR	95 % CI	I^2^	Meta-Regression *P*-Value
Study design					0.116
Cohort	14	0.81	0.70-0.94	84.6%	
C-C	7	1.10	1.05-1.15	0%	
**Study location**					0.409
Western	5	0.99	0.97-1.00	0%	
Asian	16	0.83	0.70-0.98	86.5%	
**Study setting**					0.629
Hospital based	6	0.91	0.72-1.16	0%	
Population based	15	0.86	0.77-0.95	87.3%	
**Quality of study**					0.000
Low risk of bias	13	0.73	0.65-0.83	25.3%	
Moderate risk of bias	8	1.03	0.95-1.11	70.0%	
**Multivariate adjusted analysis**					0.007
Yes	14	0.76	0.63-0.92	84.5%	
No	7	1.08	1.04-1.13	0%	
**Duration of DM**					0.577
New-onset	10	0.80	0.66-0.97	91.7%	
Less than 10 years	3	0.84	0.63-1.11	0%	
Moe than 10 years	1	1.25	0.56-2.75	-	

In subgroup analyses, the proactive association was detected in cohort studies, population-based studies, studies adjusting for covariates, Asian population, new-onset diabetic patients, and studies with low risk of bias. The subgroup analyses suggested an increased risk in case-control studies and in studies not adjusted for potential confounders. Sufficient data were not available to analyze the impact of AGI dose and duration.

Meta-analysis of RCTs revealed no significant association of AGI with cancer risk (*n* = 4 studies; OR = 0.83, 95% CI 0.20-3.46, *I*^2^ = 0%) (Figure S2).

### AGI and cancer risk for individual tumor types

**Figure 3:**
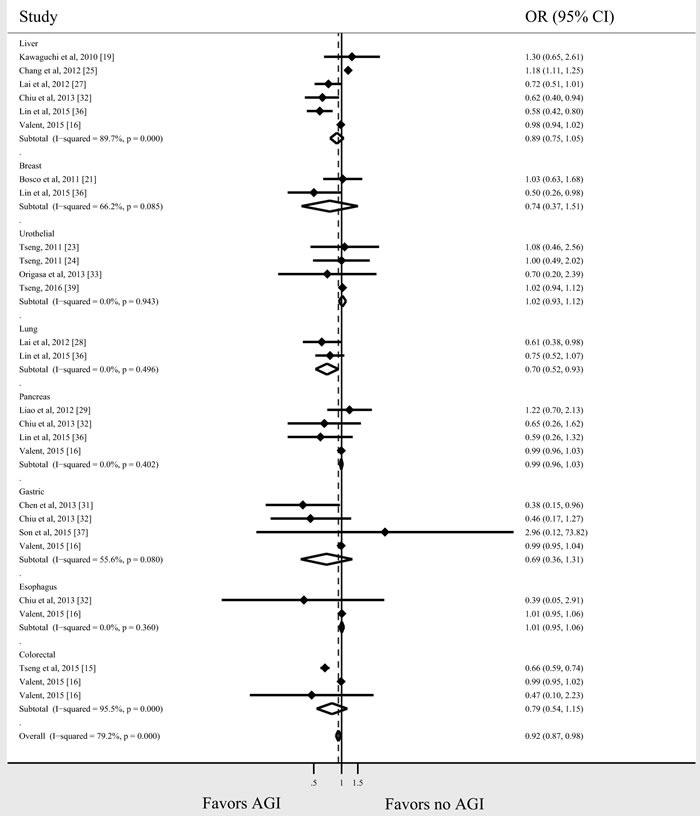
AGI and the cancer risk for individual tumor types

**Figure 4:**
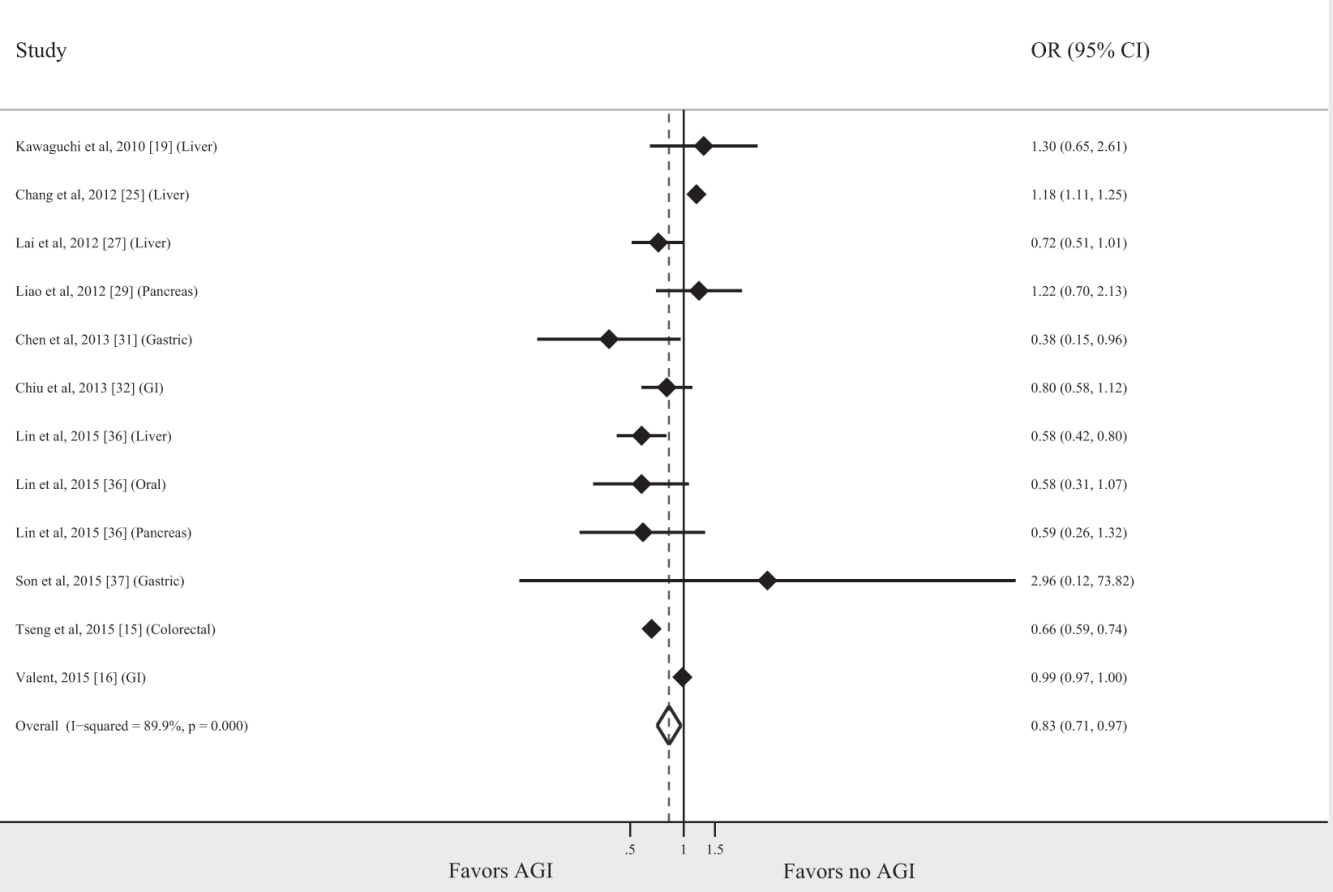
AGI and the risk for gastrointestinal cancer

Seventeen studies (16 observational studies and 1 RCT) reported the risk of cancer for individual tumor types in AGI users compared with non-users. The relationship between AGI use and risk for each tumor type is shown in Figure 3. As there were 4 Taiwanese studies on colorectal cancer from the same cohort [15, 25, 32, 36], the study with the largest cohort size was included [15]. Two studies presented data on colon and rectum cancer separately, which were pooled to derive a summary estimate for the study [16, 32]. The association between AGI use and decreased risk of cancer was most prominent in patients with lung cancer (*n* = 2 studies; OR = 0.70, 95% CI 0.52-0.93, *I*^2^ = 0%). There was a slight trend toward lower risk of colorectal, liver, gastric, and breast cancer with AGI use (OR = 0.79, 95% CI 0.54-1.15, *I*^2^ = 96%; OR = 0.89, 95.5 % CI 0.75-1.05, *I*^2^ = 89.7%; OR = 0.69, 95% CI 0.36-1.31, *I*^2^ = 55.6%; OR = 0.74, 95% CI 0.37-1.51, *I*^2^ = 66.2%, respectively); however, these associations were not statistically significant. No significant associations were identified for pancreatic, esophageal, and urothelial cancer. A meta-analysis of studies of gastrointestinal cancer (Figure 4) showed a significant association between AGI use and reduced cancer risk (OR = 0.83, 95% CI 0.71-0.97, *I*^2^ = 89.9%).

### Publication bias

There was no evidence of significant publication bias, both quantitatively (*P* = 1.0 for Begg's test, *P* = 0.116 for Egger's test) and qualitatively, on visual inspection of the funnel plot (Figure S3).

## DISCUSSION

This study showed an overall reducing effect of AGI on cancer risk, which was inconsistent with the previous meta-analysis [7]. The previous meta-analysis noted a significantly increased risk with AGI only in the case-control studies, but not in the cohort studies or RCTs [7]. In addition, the meta-analysis included only two cohort studies and omitted important recent studies on the influence of AGI on cancer risk. Furthermore, subgroup analyses were not performed. In subgroup analyses of our present analysis, the association between AGI and cancer risk was more prominent in population-based studies, studies with low risk of bias, and studies adjusted for covariates, indicating that more prospective, well-designed studies are warranted to confirm the results.

Various explanations have been provided for the association between diabetes and cancer. Metformin has been shown to possess anti-cancer property both *in vivo* and *in vitro* [9]. It has been proposed that metformin exerts its anti-cancer properties through direct effects on cancer cells, particularly through inhibition of the AMPK/mTOR pathway, and indirect effects by decreasing glucose, insulin, insulin-like growth factor 1 (IGF-1) levels, and other inflammatory factors [9].

Metformin is the only first-line oral ADM recommended by international guidelines for the treatment of type 2 diabetes [40]. AGI is another inexpensive and well-tolerated drug that has been widely used to treat DM for more than 20 years [41]. AGIs have shown better glucose-lowering effect in Asian populations than in Western populations [42], and acarbose has shown to exhibit an efficacy similar to that of metformin in China [43]. Yang et al showed that acarbose diminished insulin and glucagon concentrations while increasing GLP-1 concentration in Chinese type 2 diabetic patients [43]. A previous study also revealed that acarbose treatment reduced postprandial hyperinsulinemia [44].

Besides hypoglycemic effect, acarbose has been shown to possess anti-inflammatory and immunomodulatory effects in animal and human studies involving both Western and Asian type 2 DM patients [45–47]. Three mechanisms can be implicated for these actions. First, acarbose may regulate gut hormones. Previous studies demonstrated that acarbose use increased GLP-1 in the serum [43, 48–51]. Second, acarbose may interact with gut microbiota. A recent study found that acarbose increased the content of gut *Bifidobacterium longum* in type 2 DM patients [47], which could help to reduce intestinal inflammation [52]. Third, the unabsorbed acarbose may have an effect on the intestinal immune system by suppressing pro-inflammatory cytokine expression in the gut [53].

Owing to the known effects of AGI on the gut, it can be hypothesized that AGI may modify the risk of gastrointestinal cancer. A study of transformed cells suggested that acarbose exerts antineoplastic effect by increasing butyrate production [54], which has protective effect against colonic cancer [55]. Previous studies suggested that fecal butyrate, which is a short-chain fatty acid, is a key colonocyte nutrient and an important survival factor for colonic epithelial cells [56]. Acarbose has been found to reduce the colonic transit time and thus change the fecal concentration of bile acids, which may have protective effect against colorectal cancer [57, 58]. In addition, acarbose use has been found to be associated with increased production of GLP-1 [43, 48–51]. Previous studies indicated GLP-1 as a potent inducer of cAMP and an inhibitor of breast cancer cell proliferation [59]. A study using CT26 tumor-bearing BALB/c mice showed that GLP-1 receptor agonist treatment increased tumor apoptosis [60]. In the APC gene knockout animal model, which develops multiple intestinal adenomas, acarbose had a regressive effect on the size of gastrointestinal adenomas but did not significantly decrease the number of colonic neoplasms [61]. Most recently, a cohort study of DM patients in Taiwan showed that acarbose use reduced the risk of incident colorectal cancer in patients with diabetes in a dose-dependent manner [15]. Our analysis showed an overall reducing effect of AGI on gastrointestinal cancer risk. However, only a slight trend toward lower risk was observed in colorectal cancer, liver cancer, and gastric cancer, which was not statistically significant. It is difficult to identify the effects of AGI on gastrointestinal cancer based on retrospective studies due to confounding by indication and reverse causality. More prospective observational studies, which account for these sources of heterogeneity, would be required to truly assess the impact of AGI on the risk of gastrointestinal cancer.

Our analysis showed that AGI use was associated with a slightly decreased risk in patients with lung cancer (*n* = 2 studies; OR, 0.86; 95% CI, 0.76-0.97). The associations observed between ADMs and lung cancer were not consistent [62–64]. The precise mechanism of the observed cancer risk reduction is not clear. The results of our analysis should be interpreted with caution as only two studies were included in the analysis.

The strengths of our study include the comprehensive and simultaneous assessment of the effects of AGI on the risk modification of cancer, and findings from multiple subgroup analyses to ensure stability of the association and identify factors responsible for heterogeneity.

There were also several limitations to our meta-analysis. First, the cancer-modifying association between AGI and cancer risk were based on data from observational studies, was apparent based on the RCT cases. In observational studies, random allocation of the intervention is not done, which is necessary to test the exposure-outcome hypothesis optimally. As a result, the chemopreventive effect of AGI seen in observational studies may be an overestimate of its true effect. However, we should acknowledge that the average follow-up time in observational studies is much longer than that in RCTs, which could explain why current clinical trials of AGI for the management of DM do not demonstrate a significant effect on cancer. Second, all studies were not adjusted for the same confounders. In addition, most patients with DM in these studies were on multiple ADMs simultaneously. As a result, as compared with patients on AGI, patients “not on AGI” (the comparator group) would be more likely to be on metformin. Therefore, it is difficult to interpret whether the risk modification inferred for any one agent is real or confounded by exposures to other ADMs. In the only monotherapy, population-based observational study conducted by Chen et al to compare cancer incidence with metformin and other ADMs in patients with DM, AGI use was not found to be associated with risk of cancer [35]. The true clinical effect of AGI on cancer risk should ideally be studied by comparing patients on AGI therapy for DM with those managed by non-medical/dietary therapy over an extended period of time.

In conclusion, meta-analysis of existing studies suggests a protective association between AGI use and risk of cancer in patients with DM. As there was considerable heterogeneity across studies, future, well-designed, prospective studies are warranted to evaluate this association better.

## MATERIALS AND METHODS

This systematic review was conducted following guidance provided by the Cochrane Handbook [65] and is reported according to the Preferred Reporting Items for Systematic Reviews and Meta-Analyses (PRISMA) guidelines [66].

### Data sources, searches, and study selection

First, a systematic literature search of MEDLINE (1966 through September 30, 2016), EMBASE (1988 through September 30, 2016), and Web of Science (1993 through September 30, 2016) databases was conducted by two study investigators for all relevant articles on the association between AGI use and risk of cancer in patients with DM. The following keywords and/or corresponding MeSH terms were used: (acarbose OR voglibose OR miglitol OR alpha glucosidase inhibitor OR alpha glucoside hydrolase inhibitor OR α glucosidase inhibitor OR α glycoside hydrolase inhibitor) AND (cancer OR tumor OR neoplasm). The title and abstract of studies identified in the search were reviewed by two authors independently to exclude studies that did not answer the research question of interest. The full text of the remaining articles was examined to determine whether it contained relevant information. Next, bibliographies of the selected articles, as well as review articles on the topic were manually searched for additional articles. Third, manual search of abstracts from major endocrinology and oncology conferences (2007-2016) was performed for additional abstracts on the topic. When information available was not complete, attempts were made to contact the corresponding authors of the studies for additional information.

Studies considered in this meta-analysis were either observational studies or RCTs that met the following inclusion criteria: (1) evaluated and clearly defined exposure to AGI, (2) reported cancer outcomes in patients with DM, and (3) reported relative risks or odds ratio (OR) or provided data for their estimation. Inclusion was not otherwise restricted by study size, language, or publication type. When there were multiple publications from the same population, only data from the most comprehensive report were included. The flow diagram summarizing study identification and selection is shown in Figure 1.

### Data extraction and quality assessment

Data were independently abstracted onto a standardized form by two reviewers. The following data were collected from each study: study design, time period of study/year of publication, location/setting of the population studied, type of DM, duration of DM, age/sex of patients included, type of tumor, dose and duration of AGI use (if reported), information source of exposure ascertainment and outcome assessment, total number of persons, OR, and 95% CI with and without adjustment for confounding factors. When data on individual tumor types were reported separately, we pooled these to derive a summary estimate for the study. For all analysis, referent group was composed of patients with DM not exposed to AGI. Conflicts in data abstraction were resolved by consensus, referring back to the original article.

Quality assessment for observational studies was performed using the Newcastle Ottawa scale [67]. A score of 7-9 represents low risk of bias, 4-6 represents moderate risk of bias, and 0-3 represents high risk of bias. The quality of RCTs was assessed using a revised form of Cochrane Collaboration's tool for assessing risk of bias in randomized trials [68]. This tool focuses on the adequacy of randomization and allocation concealment procedures, blinding, and loss to follow-up. Any discrepancies were addressed by a joint reevaluation of the original article.

### Data synthesis and analysis

The primary analysis focused on assessing the relationship between AGI and risk of cancer in patients with DM. A priori hypotheses to explain potential heterogeneity in the direction and magnitude of effect among different observational studies included type of cancer, location/setting of study (Western population *vs*. Asian population; population based *vs*. hospital based), study design (case-control *vs*. cohort), quality of study (low bias *vs*. moderate bias *vs*. high bias), and whether the study was adjusted for the potential confounding factors. Because of significant differences in the design of observational studies and RCTs, data from these RCTs were analyzed and presented separately.

We used the random-effects model described by DerSimonian and Laird to calculate meta-analytic OR and 95% CI [69]. Adjusted ORs reported in studies were used for analysis to account for confounding variables. We assessed heterogeneity between study-specific estimates with the Cochran Q statistic (*P* < 0.10) and I^2^ statistic [69, 70]. Once heterogeneity was noted, between-study sources of heterogeneity were investigated using subgroup and meta-regression analyses by study characteristics (as described above). All *P* values were two-tailed. For all tests (except for heterogeneity and publication bias), a *P* value of less than 0.05 was considered statistically significant. Subgroup analysis was conducted on all relevant study characteristics regardless of statistical significance. We investigated the presence and the effect of publication bias using a combination of the Begg's test [70] and Egger's test [71]. Statistical analyses were performed using Stata 12.1 (StataCorp). An overview of the study protocol is provided in S1 Protocol.

## SUPPLEMENTARY FIGURES





## Abbreviations

ADM: anti-diabetic medication
AGI: alpha-glucosidase inhibitor
DM: diabetes mellitus
GLP-1: glucagon-like peptide-1
RCT: randomized controlled trial
