# Supplementary material for: Alpha-glucosidase inhibitors and risk of cancer in patients with diabetes mellitus: a systematic review and meta-analysis
Source: Oncotarget. 2017 Apr 28;8(46):81027–39. doi: 10.18632/oncotarget.17515 (PMC5655259; doi:10.18632/oncotarget.17515)
Supplement: Supplementary file 1 [file oncotarget-08-81027-s001.pdf]

Alpha-glucosidase inhibitors and risk of cancer in patients with diabetes mellitus: a systematic review and meta-analysis

SUPPLEMENTARY MATERIALS

S1 Protocol

Study protocol for systematic review and meta-analysis to determine the relation between alpha-glucosidase inhibitors (AGI) and the risk of cancer

"Alpha-Glucosidase Inhibitors and Risk of Cancer in Patients with Diabetes Mellitus: A Systematic Review and Meta-Analysis"

Objective

In this systematic review and meta-analysis, we aim to investigate the relationship between AGI and cancer risk in patients with diabetes mellitus (DM).

Inclusion criteria

Study type

All study types will be eligible to enter meta-analysis.

We will include all studies which were published as original report and present information on the relation between AGI and cancer.

Participants

Patients with DM will be included.

We will perform sub-group analyses (e.g. different type of cancer, study design, location, etc.).

Definition of exposition

All studies which reported information on AGI will be included as well as studies which reported odds ratios (OR) and 95% confidence intervals (95% CI) (or data with which to calculate them) for risk of cancer.

Outcome variable

All studies which reported risk of cancer will be included.

Characteristics of studies and the type of cancer will be stratified.

Outcome measures

The OR will either be extracted from the published article or calculated by the authors.

If the OR is not directly reported or cannot be readily extracted from the published data, the reviewers will contact the corresponding authors for additional information.

Publication type

Full published papers will be eligible (language restrictions were applied to English).

Search Methods

We will search the following electronic databases:

MEDLINE (via PubMed) (1966 through September 30, 2016)

EMBASE (1988 through September 30, 2016)

Web of Science (1993 through September 30, 2016)

In these databases, we will search according to the thesaurus of the NCBI MESH browser the following terms and combinations of keywords in full text:

The following keywords will be employed:

1. “acarbose”
2. “voglibose”
3. “miglitol”
4. “alpha glucosidase inhibitor”
5. “alpha glucoside hydrolase inhibitor”
6. “α glucosidase inhibitor”
7. “α glycoside hydrolase Inhibitor”
8. “cancer”
9. “tumour”
10. “neoplasm”
11. “(1) OR (2) OR (3) OR (4) OR (5) OR (6) OR (7)” AND “(8) OR (9) OR (10)”

Additionally, bibliographies of identified publications and published reviews will be hand searched for potentially relevant articles. Authors will be contacted if data, methods and/or parameter definitions provided from the respective studies are unclear.

Reviews

All references cited in the identified reviews will be manually searched for potentially relevant studies.

Data collection

Two reviewers (ZYM, LHY) will independently scrutinize the list of titles, and if available the abstracts, to determine potential usefulness of the article. Final selection will be based on the full text of potentially relevant articles by the two reviewers independently. In cases of disagreement, both authors will review the materials together until a consensus is reached. Study quality will be measured using the Newcastle–Ottawa scale [1].

The following study characteristics will be extracted: study design, time period of study/year of publication, location/setting of the population studied, type of DM, duration of DM, age/sex of patients included, type of tumor, dose and duration of AGI use (if reported), information source of exposure ascertainment and outcome assessment, total number of persons, OR, and 95% confidence intervals (CI) with and without adjustment for confounding factors.

From all eligible studies, relevant data will be abstracted in duplicate, using a standardized data extraction sheet. An independent reviewer will confirm all data entries and will check at least twice for completeness and accuracy.

Meta-analysis & Meta-regression

Dichotomous comparisons

Data on numbers of subjects with and without cancer through AGI and corresponding crude odds ratios and 95% confidence intervals will be calculated.

Random-effects models to estimate the pooled odds ratios for risk of cancer due to exposure to AGI will be constructed across all studies [2].

Assessment of heterogeneity

Impact of heterogeneity will be assessed by calculating the Cochran Q statistic ($p < 0.10$) and the I^2 [2, 3].

Subgroup/Sensitivity analyses

To identify potential sources of heterogeneity and sources of bias, studies will be stratified by study characteristics and the type of tumour.

Meta-regression was used to evaluate whether effect size estimates were significantly different by specific study characteristics and quality factors. Meta-regression p-values were provided.

Evaluation of bias and confounding

Publication bias

Publication bias will be assessed by a combination of the Begg's test [4] and Egger's test [5] quantitatively, and qualitatively, by visual inspection of the funnel plot.

Discussion and Evaluating

The results will be critically and integratively discussed.

REFERENCES

1. Wells G, Shea B, O'Connell D, Peterson J, Welch V, Losos M, Tugwell P. The Newcastle-Ottawa Scale (NOS) for assessing the quality of nonrandomized studies in meta-analyses. 2013. http://www.ohri.ca/programs/clinical_epidemiology/oxford.asp
2. DerSimonian R, Laird N. Meta-analysis in clinical trials. Control Clin Trials. 1986; 7:177–88.
3. Higgins JP, Altman DG, Gøtzsche PC, Jüni P, Moher D, Oxman AD, Savovic J, Schulz KF, Weeks L, Sterne JA, and Cochrane Bias Methods Group, and Cochrane Statistical Methods Group. The Cochrane Collaboration's tool for assessing risk of bias in randomised trials. BMJ. 2011; 343:d5928.
4. Higgins JP, Thompson SG. Quantifying heterogeneity in a meta-analysis. Stat Med. 2002; 21:1539–58.
5. Egger M, Davey Smith G, Schneider M, Minder C. Bias in meta-analysis detected by a simple, graphical test. BMJ. 1997; 315:629–34.

Search Strategy

MEDLINE (PubMed), Embase and, Web of Science articles published in English before September 30, 2016 were identified. Electronic search was performed independently by two Authors and supplemented by scanning reference lists of all relevant articles, including reviews, by hand searching of relevant journals. We used terms related to exposure and outcomes.

Exposure: acarbose OR voglibose OR miglitol OR alpha glucosidase inhibitor OR alpha glucoside hydrolase inhibitor OR α glucosidase inhibitor OR α glycoside hydrolase Inhibitor
Outcome: cancer OR tumor OR neoplasm

For PubMed the search was

(acarbose OR voglibose OR miglitol OR (alpha glucosidase inhibitor) OR (alpha glucoside hydrolase inhibitor) OR (α glucosidase inhibitor) OR (α glycoside hydrolase Inhibitor)) AND (cancer OR tumor OR neoplasm)
Search strategy was specifically translated for each database

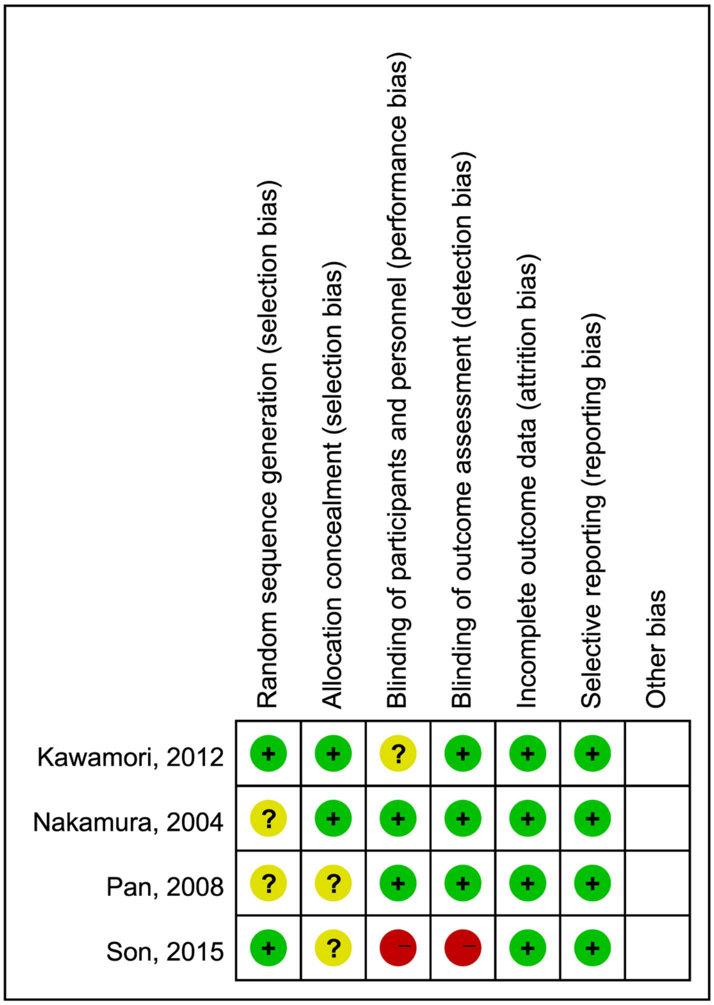

Figure S1. Risk of bias assessment in randomized controlled trials

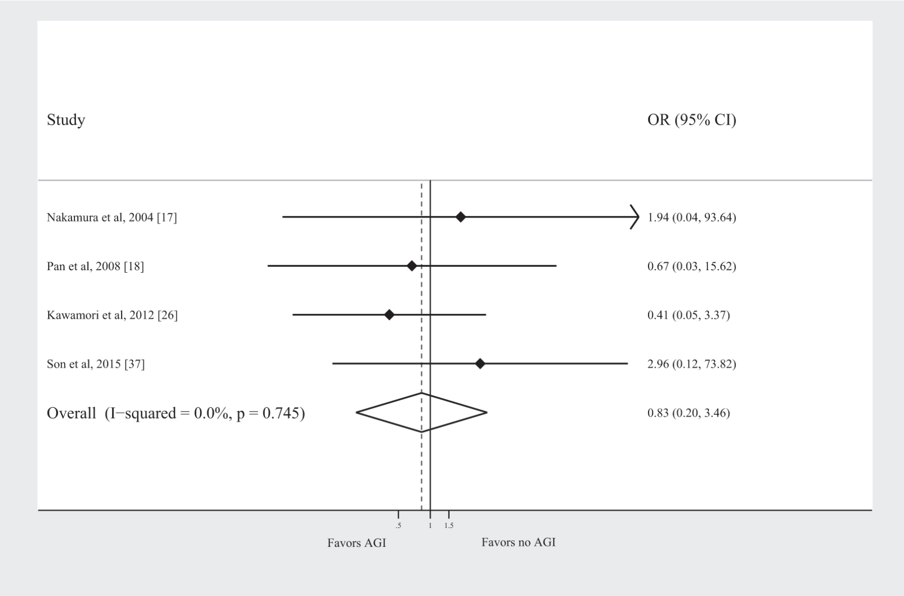

Figure S2. Risk of bias assessment in randomized controlled trials

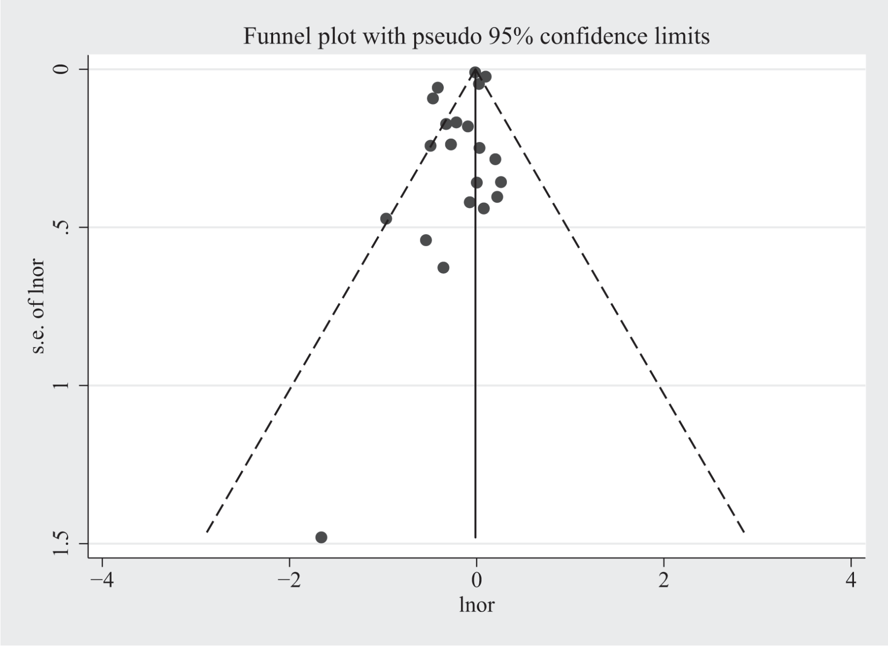

Figure S3. Risk of bias assessment in randomized controlled trials
